# Internalized HIV/AIDS-related Stigma in a Sample of HIV-positive People in Bangladesh

**DOI:** 10.3329/jhpn.v30i1.11272

**Published:** 2012-03

**Authors:** Md. Tanvir Hasan, Samir Ranjan Nath, Nabilah S. Khan, Owasim Akram, Tony Michael Gomes, Sabina F. Rashid

**Affiliations:** ^1^James P. Grant School of Public Health, BRAC University, Mohakhali, Dhaka 1212, Bangladesh (*Deceased); ^2^Research and Evaluation Division, BRAC, Mohakhali, Dhaka 1212, Bangladesh; ^3^Plan International, Bangladesh, Dhaka, Bangladesh; ^4^UNAIDS, Bangladesh, Dhaka, Bangladesh

**Keywords:** Acquired immunodeficiency syndrome, Discrimination, Human immunodeficiency virus, Stigma, Bangladesh

## Abstract

Internalized stigma among people living with HIV/AIDS (PLHA) is prevalent in Bangladesh. A better understanding of the effects of stigma on PLHA is required to reduce this and to minimize its harmful effects. This study employed a quantitative approach by conducting a survey with an aim to know the prevalence of internalized stigma and to identify the factors associated with internalized stigma among a sample of 238 PLHA (male=152 and female=86) in Bangladesh. The findings suggest that there is a significant difference between groups with the low and the high-internalized HIV/AIDS stigma in terms of both age and gender. The prevalence of internalized stigma varied according to the poverty status of PLHA. An exploratory factor analysis (EFA) found 10 of 15 items loaded highly on the three factors labelled self-acceptance, self-exclusion, and social withdrawal. About 68% of the PLHA felt ashamed, and 54% felt guilty because of their HIV status. More than half (87.5% male and 19.8% female) of the PLHA blamed themselves for their HIV status while many of them (38.2% male and 8.1% female) felt that they should be punished. The male PLHA more frequently chose to withdraw themselves from family and social gatherings compared to the female PLHA. They also experienced a higher level of internalized stigma compared to the female PLHA. The results suggest that the prevalence of internalized stigma is high in Bangladesh, and much needs to be done by different organizations working for and with the PLHA to reduce internalized stigma among this vulnerable group.

## INTRODUCTION

From the beginning, since its first diagnosis, HIV/AIDS has been recognized as a disease associated with fear and stigma (1). Stigma, one of the greatest barriers to preventing new infections of HIV, is a major cause hindering efforts in HIV/AIDS-prevention programmes, HIV testing, and access to treatment (1,2). Although stigma is the major significant barrier, little empirical research has been conducted on this issue.

HIV/AIDS-related stigma is a complex multidimensional issue that varies from individual to individual because of the different perspectives about the disease. People living with HIV/AIDS (PLHA) are not different in this aspect.

Goffman defined stigma as a powerful social label, stemming from a discrediting attribute of the individual, which radically changes their social identity (3). Several other authors have divided stigma into two kinds: ‘felt’ stigma, also known as ‘perceived’ or ‘internal’ stigma, and ‘external’ or ‘enacted’ stigma (4-8). According to Brown *et al.*, ‘felt’ or ‘perceived’ or ‘internal’ stigma refers to real or imagined fear of societal attitudes and potential discrimination arising from a particular undesirable attribute, disease (such as HIV), or association with a particular group or behaviour (e.g. homosexuality and promiscuity) (5). ‘Enacted’ or ‘external’ stigma, on the other hand, refers to the actual experience of discrimination (6,8). The two kinds of stigma are interlinked. For example, people experience discrimination because of stigma; discrimination leads to internal stigma; and internal stigma again reinforces and legitimizes stigma (9). Of the two kinds of stigma, internalized stigma receives less attention from researchers and programme planners (4).

Since HIV/AIDS-related internalized stigma has an impact on the well-being of PLHA and can hinder his/her participation in most community and social activities, this study focused on measuring internalized stigma among a group of diverse PLHA in Bangladesh. In our study, internalized stigma was defined as the product of internalization of shame, blame, hopelessness, guilt, and fear of discrimination associated with being HIV-positive (4).

Results of studies showed that internalized stigma may discourage PLHA from seeking care (7) and may increase the levels of their loneliness compared to the general population (10,11). PLHA who feel stigmatized also feel anxiety, depression, and alienation, and these feelings of anxiety, depression, and alienation are associated with disruptions in normal social relationships (12). These findings describe the crucial effects of internalized HIV/AIDS-related stigma on the mental health of PLHA and suggest the need for conducting more research on this issue. Gender relationships also play an important role in experiencing stigma. Studies have documented women's vulnerability of experiencing HIV/AIDS-related stigma and discrimination (13,14).

Historically, Bangladesh is a patriarchal society where men exercise control over women's sexua-lity and their access to services. Men tend to be the main decision-makers within the family, and the social norms and responsibilities allow men to control women's behaviour. As a result of gender inequality and social structure, women living with HIV/AIDS are more at risk of experiencing stigma.

Although the prevalence of HIV/AIDS in Bangladesh remains low, the number of PLHA has been steadily rising. According to the 2010 United Nations General Assembly Special Session (UNGASS) country report, 1,745 HIV cases were detected in Bangladesh, and 250 new cases were detected in 2009 (15). While the number of PLHA has been increasing day by day, research on HIV/AIDS stigma has so far been limited. An extensive review of literature found no published articles focusing on internalized stigma among PLHA in Bangladesh. This is a pioneering study in Bangladesh, which focuses on internalized stigma among the PLHA. However, some studies focused on stigmatized attitudes of different individuals towards PLHA. A study on the stigmatized attitudes of healthcare workers towards PLHA found that healthcare support staff had highly stigmatized attitudes, followed by medical technicians and nurses (16). In a study on knowledge about and attitudes towards belief and practice (KABP) relating to HIV/AIDS among Bangladeshi overseas job-seekers, 28.6% of workers (n=300) reported that they would not want to continue living with an HIV-positive family member; 16.7% felt that the PLHA should not receive health services like other patients; and 95% reported that they would not want to work with PLHA (17). In another study on knowledge about and attitudes towards HIV/AIDS among icddr,b staff, Islam *et al*. found that 41% of 293 staff members surveyed believed that PLHA should not be allowed to work (18). Results of these three studies showed a high prevalence of HIV/AIDS-related stigma embedded in the society and suggest that there is an urgent need for research on issues relating to HIV/AIDS-related stigma to highlight the impact of such stigma.

This study highlighted the present picture of the prevalence of HIV/AIDS-related internalized stigma among PLHA in Bangladesh. It also identified the domains that might be helpful to discriminate against different manifestations of internalized HIV/AIDS-related stigma.

## MATERIALS AND METHODS

We conducted a quantitative survey of 238 adult PLHA (aged not less than 15 years) following the stigma index questionnaire developed by the International Planned Parenthood Federation (IPPF) in partnership with the Joint United Nations Programme on HIV/AIDS (UNAIDS), the Global Network of People Living with HIV (GNP+), and the International Community of Women Living with HIV (ICW). Data were collected during October 2008–November 2008 in four divisions: Dhaka, Chittagong, Sylhet, and Khulna, using the face-to-face interview technique. The participants (n=238) were recruited from four organizations, such as Ashar Alo Society, Mukto Akash Bangladesh, GEON Health Foundation, and CAAP, working for and with the PLHA and giving care, treatment, and support to them. First, these four organizations were requested to provide the names and details of 405 members living in the four divisions. The list of members well-represented the PLHA of the four divisions as it included most adult PLHA aged 15 years and above, living in these areas. From this list of 405 members, a systematic random sample of 278 PLHA was taken. However, we were able to conduct 238 interviews from the list of 278 participants because many participants were unavailable at the time of interview, and some of them did not agree to participate.

### Internalized stigma scale

To find the prevalence of internalized HIV/AIDS-related stigma among men and women, an internalized stigma scale was developed. The internalized HIV/AIDS-related stigma scale included 15 questions from the section “Internal stigma (the way you think about yourself) and your fear” of the stigma index questionnaire. These 15 items cover three domains of internalized stigma (self-acceptance, self-exclusion, and social withdrawal) and were adapted from the set of internal stigma indicators reported by Brouard and Wills (4). Every item had a two-option response format: ‘Yes’ or ‘No’, which were numerically coded as 1 for ‘Yes’ and 0 for ‘No’. For example, for the question, “In the last 12 months are you feeling ashamed of your HIV status?”, a PLHA was categorized as internally stigmatized if he/she answered ’Yes’.

For every question, a score was given, and these individual scores were added to obtain the cumulative score. Finally, the cumulative score was used for determining the extent of internalized stigma ranging from 0 to 15. A cumulative frequency curve for the internalized stigma score variable was used for finding the cut-off points to classify the individuals into different groups. The high-internalized stigma group included those individuals with score greater than or equal to the value of the third quartile, ranging from 6 to 11 on the internalized stigma score variable while the low-internalized stigma group included those individuals with score lower than or equal to the first quartile, ranging from 0 to 3 on the internalized stigma score variable. This method was originally adapted from measures developed by Lee *et al.* (19), where a subset of participants (HIV-positive men and women) was classified into a highor a low-internalized stigma group. Individuals with the upper one-third of the scores were defined as a high-internalized stigma group, and individuals with the lower one-third of the scores were defined as a low-internalized stigma group.

### Poverty and internalized stigma

To examine whether the level of internalized stigma varies according to the poverty status of PLHA, we used the concept of poverty. The poverty-lines are basically of two types: (a) National poverty-line generally set up by the national government and (b) An international poverty-line, such as a ‘US$ 1-a-day’ poverty-line. The percentage of people who are living under the ‘US$ 1-a-day’ poverty-line were said to be hardcore poor (20).

An international poverty-line (US$ 1-a–day poverty-line) was used for assessing the current poverty situation among the PLHA. The purchasing power parity (PPP) exchange rate method (20) was used for determining the equivalent US$ 1-a-day international poverty-line and for converting these values in local currency. For Bangladesh, the cut-off point was found to be Tk 1,268 at October 2008 prices. Therefore, any individual who had a monthly income of less than or equal to Tk 1,268 was categorized as hardcore poor. The proxy individual income of the PLHA has been derived by dividing the household's total income by the number of adult members of that particular house.

### Analysis of data

Data were analyzed using the SPSS software (version 11.5) (SPSS Inc., Chicago, IL). We assessed the prevalence of internalized stigma among the PLHA according to gender and poverty status.

To examine whether the prevalence of internalized stigma varies across the sample, we first used the frequencies of responses of the internalized stigma items. We then obtained the cumulative internalized stigma score for each of the PLHA and cate-gorized these into groups. Finally, we conducted several chi-square tests to examine the possible differences in the two groups (high-internalized stigma group and low-internalized stigma group) according to a number of characteristics (Table 1).

In search of finding the possible domains of internalized stigma linked to HIV/AIDS, the original 15 items of the internalized stigma scale were assessed by conducting exploratory factor analysis using the principal components extraction method and oblique factor rotation with Scree plot.

### Ethical approval

At the beginning of each interview, written informed consent was obtained from each participant. The ethical review committee of the James P. Grant School of Public Health, BRAC University, Dhaka, Bangladesh, approved the study.

## RESULTS

### Demographic characteristics of PLHA

Table 2 shows the sociodemographic characteristics of the study PLHA. About three-fourths (72.7%) of the 238 PLHA were living in small towns or villages while 27.3% were living in large towns or cities at the time of interview. Nearly 64% of the PLHA were male (n=152); 36% were female (n=86); 46.2% were aged 30-39 years; and 24.4% were aged 40-49 years. Only 11.3% of the PLHA had their technical college/university-level education whereas about 23% had no educational degree. More than 35% of them were unemployed.

### Prevalence of internalized stigma

Table 3 shows the prevalence of internalized stigma among the 238 PLHA. The percentage of PLHA feeling guilty was two times higher among the males than among the females. Most (about 88%) males blamed themselves for being HIV-positive while this result was found reversely true for the females (20%). The percentage of PLHA with low self-esteem was also higher among the males (61.2%) than among the females (38.4%). Many (38.2%) male PLHA felt that they should be punished while this rate was comparatively low for the females (8.1%). Because of the internalized stigma, 11.2% of the male and 5.8% of the female PLHA decided not to attend any social gathering. Many of them (17.1% of male PLHA and 16.3% of female PLHA) decided not to visit a hospital even when they were required to go there for treatment. The prevalence of HIV/AIDS-related internalized stigma also varied according to the poverty status of PLHA. The percentage of the PLHA who decided not to apply for a job and to stop working was higher among the non-poor group than among the poor group (Table 3).

**Table 1.** Comparison of categorical variables: PLHA with a low-level versus high-level of internalized stigma

Variable	Low internalized stigma (n=82)	High internalized stigma (n=84)	Chi-square value	p value
Age (years)				
15-24	12.2	3.6		
25-29	25.6	8.3		
30-39	42.7	52.4	16.931	0.002*
40-49	14.6	31.0		
50+	4.9	4.8		
Male	50.0	78.6	14.78	<0.001*
Poor	28.0	19.7	1.87	0.171
Area of residence				
Very remote area	7.3	9.5		
Small village or town	64.6	66.7	0.554	0.758
Big city	28.0	23.8		
Education				
No formal education	20.7	25.0		
Primary	24.4	32.1	2.468	0.481
Secondary	40.2	32.1		
College/university	14.6	10.7		
Occupational status				
Full-time employee	18.3	17.9		
Part-time employee	3.7	6.0		
Full-time self-employed	14.6	16.7	7.745	0.101
Part-time self-employed	18.3	25.0		
Unemployed	45.1	26.2		
HIV (years)				
0-<1	22.0	16.7		
1-4	52.4	48.8	2.278	0.517
5-9	20.7	25.0		
10 and above	4.9	9.5		
Marital status				
Married	78.0	61.9		
Unmarried	6.1	13.1	5.350	0.069
Divorced or widowed	15.9	25.0		

*Significant at 1% level;

PLHA=People living with HIV/AIDS

### Low-level versus high-level of internalized HIV/AIDS stigma in relation to several characteristics

As described in the methodology section, we classified a subset of the participants into two groups (low-internalized stigma group and high-internalized stigma group) according to their scores. We found that 82 (34.4%) PLHA fell in the low-internalized stigma group and 84 (35.3%) PLHA fell in the high-internalized stigma group. Table 1 shows the results of the chi-square test for the two groups (high-internalized stigma group and low-internalized stigma group) for a number of characteristics. The results of the chi-square test showed no significant differences between the high and the low-internalized stigma groups according to duration with HIV in years, marital status, level of education, employment, area of residence, and poverty status. However, there were significant differences between the two groups in age and gender. The participants aged 30-39 years were found more likely to experience high-internalized stigma compared to any other group. The male PLHA were more likely to experience high-internalized stigma compared to the female PLHA.

**Table 2.** Sociodemographic characteristics of study participants

Characteristics	No.	%
Age (years)		
15-19	1	0.4
20-24	17	7.1
25-29	38	16.0
30-39	110	46.2
40-49	58	24.4
50+	14	5.9
Total	238	100
Gender		
Male	152	63.9
Female	86	36.1
Total	238	100
Level of education		
No formal education	54	22.7
Primary completed	66	27.7
Secondary completed	91	38.2
Technical college/university	27	11.3
Total	238	100
Employment		
Full-time employee	46	19.3
Part-time employee	9	3.8
Working full-time but not as employee	49	20.6
Doing causal part-time work	50	21.0
Unemployed and not working at all	84	35.3
Total	238	100
Area of residence		
Large town or cities	65	27.3
Rural villages, including small town	173	72.7
Total	238	100
Categories*		
Gays or lesbians	4	1.7
Sex workers	3	1.3
Injecting drug-users	22	9.2
Refugee/floating people	1	0.04
Internal migrants	8	3.4
Migrant workers	85	35.7
Prisoners	16	6.7
None of the above	119	50.0

*Multiple responses

### Factors linked to internalized stigma

The data-screening process of factor analysis was done by, first, finding the correlation matrix. The results of the correlation matrix ensured that none of the correlation coefficients is zero, and no extreme multi-collinearity exists. The initial factor analysis solution (oblique rotation using direct oblimin procedure) yielded five factors with eigenvalues greater than 1. However, the results suggested dropping of five items having low factor loadings (standard regression coefficient <0.30) from the 15 items. Finally, 10 items were used for the analysis. Both Scree plot and Kaiser's eigenvalue greater than 1 method (21) confirmed the three existing underlying factors. A factor was defined by including those items that have a factor loading of >0.40 and did not load on multiple factors. The pattern matrix was used for interpreting the factors. The factor loadings are presented in Table 4.

The three factors that emerged from the exploratory factor analysis were ‘self–acceptance’, ‘self-exclusion’, and ‘social withdrawal’. Five items (1, 2, 3, 4, and 5) loaded on the first factor ‘self-acceptance’ and included the questions relating to feeling ashamed, feeling guilty, blaming self, have low self-esteem, and desiring punishment. The second set of factor loadings (6,7, and 8) were included on the second factor ‘self-exclusion’ and was related to the decision the PLHA made because of their HIV status. This factor included the questions on decision of not to work, decision of not to apply for a job, and decision of not to participate in any educational programme. Two items (9 and 10) loaded on the third factor ‘social withdrawal’ and included the questions relating to their decision of not to get married and not to have sex.

## DISCUSSION

Internalized stigma was prevalent among the study participants and varied according to their gender and poverty status. Every three in four male PLHA felt ashamed because of their HIV status. The proportion of PLHA who felt to be punished for their HIV status was four times higher among the males than among the females. The males frequently chose not to attend any family and social gathering compared to the females. These findings suggest that the male PLHA more frequently suffered from internalized stigma than the female PLHA. These findings are consistent with the findings of a study by Simbayi *et al*. that men are more likely to report experiencing HIV/AIDS-related internalized stigma than women (22). However, we must be cautious when interpreting this result. Men frequently experience stigma perhaps because traditionally they have more freedom and mobility which allows them to engage in marital and sexual affairs. They are the main decision-makers within the family and usually control women's sexual life and their choices. As a result, men seem to blame themselves for becoming infected and feel ashamed for their past activities, such as drug-use or involvement in unprotected sex, with promiscuity highlighted as a main cause for HIV/AIDS. On the other hand, women have little control over their body and choice of marriage partners. They also have little access to services. Consequently, they may see themselves as victims, with little choice.

**Table 3.** Prevalence of internalized stigma according to gender and poverty status of PLHA

Variable	Male (n=152)	Female (n=86)	Hardcore poor (n=59)	Non-poor (n=179)
I feel ashamed	75.0	60.5	72.9	68.7
I feel guilty	72.4	36.0	50.8	62.0
I blame myself	87.5	19.8	52.5	66.5
I have low self-esteem	61.2	38.4	47.5	54.7
I feel I should be punished	38.2	8.1	15.3	31.3
I feel suicidal	23.0	17.4	15.3	22.9
I have chosen not to attend social gathering(s)	11.2	5.8	6.8	10.1
I have isolated myself from my family and/or friends	7.9	3.5	1.7	7.8
I took decision to stop working	2.0	3.5	-	3.4
I decided not to apply for a job/work or for a promotion	7.2	15.1	3.4	12.3
I withdrew from education/training or did not take up an opportunity for education/training	3.3	3.5	1.7	3.9
I decided not to get married	71.1	87.2	81.4	75.4
I decided not to have sex	7.9	37.2	22.0	17.3
I avoided going to a local clinic when I needed to	21.7	20.9	23.7	20.7
I avoided going to a hospital when I needed to	17.1	16.3	16.9	16.8

PLHA=People living with HIV/AIDS

**Table 4.** Exploratory factor analysis pattern matrix loadings for 10 HIV internalized stigma scale items

Item	Factors		
	Self-acceptance	Self-exclusion	Social withdrawal
I feel ashamed	0.707	−0.127	0.008
I feel guilty	0.671	0.050	−0.280
I blame myself	0.471	0.153	−0.519
I have low self-esteem	0.806	−0.030	0.062
I feel I should be punished	0.675	0.055	0.141
I took the decision to stop working	−0.072	0.720	0.024
I decided not to apply for a job/work or for a promotion	119	0.739	0.155
I withdrew from education/training or did not take up an opportunity for education/training	−0.071	0.841	−0.126
I decided not to get married	0.110	−0.035	0.752
I decided not to have sex	−0.027	0.151	0.700

The results of the study revealed that the PLHA who were not poor more frequently chose to stop working and not to apply for a job/work compared to the poor. This is perhaps because the non-poor have some resources to cope up without an income. The results of bivariate analysis revealed no significant differences in the poverty status between the two groups. This finding suggests that poverty may not be a potential factor that increases internalized stigma among PLHA. However, to some extent, this result is unexpected because the poor are socially and economically disadvantaged within communities and lack access to basic services, including education and treatment. Therefore, they are more at a risk of experiencing HIV/AIDS-related stigma. Thus, more research is needed to see the effects of poverty on internalized stigma.

Stigma continues to be one of the main barriers that prohibit PLHA from seeking healthcare services. The results of the present study suggest that many PLHA decided not to visit a local clinic or a hospital even when it was necessary for them to obtain medicines and treatment for various ailments. These findings are consistent with the findings of several other studies that internalized stigma may discourage PLHA from seeking treatment (4,23). Alternatively, PLHA may have decided not to visit a hospital or a clinic in fear of experiencing potential discrimination from healthcare workers. These results emphasize the need for improving knowledge of healthcare providers through the implementation of stigma-related programmes.

There was a significant difference in age and gender between the low and the high-internalized stigma group. Of the PLHA who were aged 30-39 years, the males were more likely to experience a higher level of internalized stigma. There was no significant difference in the duration (in years) of HIV between the two groups. This finding is different from the finding of a study by Lee *et al*., indicating that a high-internalized stigma group was diagnosed more recently (19). In another study in the United States, Vanable *et al*. also found time since diagnosis as positively associated with having experienced discrimination (24).

The exploratory factor analysis identified three domains (self-acceptance, self-exclusion, and social withdrawal) that might be helpful to discriminate against different manifestations of internalized HIV/AIDS stigma. The factor self-acceptance relates to feelings of PLHA. This factor might be useful in identifying the needs of PLHA. For example, PLHA who have a low level of self-acceptance require more counselling and psychosocial support. The factor ‘self-exclusion’ relates to the situation when a person living with HIV/AIDS excludes himself/herself from receiving services and availing of the opportunities of society (4). The results of our study revealed a positive relationship between the factors—self-acceptance and self-exclusion. PLHA who have a low level of self-acceptance of their HIV status are more at a risk of self-exclusion. The other factor—social withdrawal—refers to the self-imposed isolation that led PLHA to exclude themselves from sexual and loving relationships (4). This factor negatively correlated with the factor—self-acceptance, meaning that PLHA with low levels of self-acceptance can have high social withdrawal. Some findings of our study on exploratory factor analysis are consistent with the findings of a study by Sayles *et al*., who viewed HIV/AIDS-related internalized stigma as multidimensional (25). Although we found these three factors to be extremely important to describe the context of internalized stigma, future research should include more items on internalized stigma to describe all the factors linked to internalized stigma

### Limitations

The present study had several limitations. We were only able to recruit our participants (although randomly sampled) through the existing PLHA peer group network. Thus, the sample is not completely representative of the national population living with HIV/AIDS in the country. Another limitation was that individual income of the PLHA was not reported in the data. To examine the poverty status of PLHA, we used the average income of each member of the house (gross household income divided by total adult family members in that house). We also acknowledge that the study measured only a few domains of internalized stigma, and other domains reported in the literature include over-compensation, fear of disclosure, and subterfuge (4). The study was also limited in the sense that it did not fully explore as to why levels of internalized stigma varied among the sample participants and what the factors were that increased or decreased the levels of internalized stigma experienced by them.

### Conclusions

Even with its limitations, the study is pioneering as it attempts to know the prevalence of internalized stigma and the factors linked to internalized stigma among a sample of 238 PLHA in Bangladesh for the first time. Based on the findings, we conclude that internal stigma is very common among all PLHA, and most of them feel some form of stigma in their life. The silence surrounding this virus and disease, along with the misconceptions and widely-perceived negative risk factors associated with HIV/AIDS (e.g. promiscuity, drug-use) among both PLHA and general population, might be the potential cause of internalized stigma. It is clear from the findings that internalized stigma affects the psychological well-being of PLHA. Although it is the key issue that prohibits PLHA from participating in different social activities, national-level intervention to reduce stigma among the PLHA is yet to be taken. To combat stigma among the PLHA, comprehensive programmes, including education to understand complexities and factors which surround HIV/AIDS, can work towards reducing its stigmatization. Support groups/network, including PLHA and other stakeholders, might also help reduce stigma at the individual level. More mass-media campaign on HIV/AIDS, its causes, knowledge, and prevention and moving away from just a negative focus on risks/blaming of individuals, may work towards encouraging compassion, understanding, and reducing stigma which is deeply embedded in the society.

## ACKNOWLEDGEMENTS

The study received financial support from the Joint United Nations Programme on HIV/AIDS. The authors gratefully acknowledge the continuous support and assistance of members and staff of the four organizations working for and with the PLHA: Ashar Alo Society, Confidential Approach to AIDS Prevention, GEON Health Foundation, and Mukto Akash Bangladesh. They also acknowledge the support from the International Planned Parenthood Federation. The authors thank Mr. Dan Odallo, Country Coordinator, UNAIDS, Bangladesh, and Dr. Tahmina Mirza, Director, AIDS, Family Planning Association of Bangladesh, for their continuous support during the study. They also thank Mr. Al-Mahmud, System Administrator, James P. Grant School of Public Health, for helping them in formatting the tables and text.

## References

[1] Parker R, Aggleton P (2003). HIV and AIDS-related stigma and discrimination: a conceptual framework and implications for action. Soc Sci Med.

[2] Aggleton P, Parker R (2002). World AIDS campaign 2002-2003: a conceptual framework and basis for actions: HIV/AIDS stigma and discrimination.

[3] Goffman E (1963). Stigma: notes on the management of spoiled identity.

[4] Brouard P, Wills C (2006). A closer look: the internalisation of stigma related to HIV.

[5] Brown L, Macintyre K, Trujillo L (2003). Intervention to reduce HIV/AIDS stigma: what have we learned?. AIDS Educ Prev.

[6] Jacoby A (1994). Felt versus enacted stigma: a concept revisi-ted. Evidence from a study of people with epilepsy in remission. Soc Sci Med.

[7] Malcolm A, Aggleton P, Bronfman M, Galvao J, Mane P, Verrall J (1998). HIV-related stigmatization and discrimination: its forms and contexts. Crit Public Health.

[8] Scambler G (1998). Stigma and disease: changing paradigms. Lancet.

[9] Morrison K (2006). Breaking the cycle: stigma, discrimination, internal stigma and HIV.

[10] Vance DE (2006). Self-rated emotional health in adults with and without HIV. Psychol Reps.

[11] Ware NC, Wyatt MA, Tugenberg T (2006). Social relationships, stigma and adherence to antiretroviral therapy for HIV/AIDS. AIDS Care.

[12] Crandall CS, Coleman R (1992). AIDS related stigmatization and the disruption of social relationships. J Soc Personal Relationship.

[13] de Bruyn M (1992). Women and AIDS in developing countries. Soc Sci Med.

[14] Ingstad B (1990). The cultural construction of AIDS and its consequences for prevention in Botswana. Med Anthropol Q.

[15] Bangladesh (2010). Ministry of Health and Family Welfare. Directorate General of Health Services. National AIDS/STD Programme. 2010 UNGASS country progress report—Bangladesh; reporting period: January 2008–December 2009.

[16] Hossain MB Kippax S Stigmatised attitudes toward people living with HIV in Bangladesh: health care workers perspectives. Asia Pacific J Public Health 2009. (doi: 10.1177/1010539509346980). 10.1177/1010539509346980 19825839

[17] Rahman M, Shimu TA, Fukui T, Shimbo T, Yamamoto W (1999). Knowledge, attitudes, beliefs and practices about HIV/AIDS among the overseas job seekers in Bangladesh. Public Health.

[18] Islam MT, Mostafa G, Bhuiya AU, Hawkes S, de Francisco A (2002). Knowledge on, and attitude towards, HIV/AIDS among staff of an international organization in Bangladesh. J Health Popul Nutr.

[19] Lee RS, Kochman A, Sikkema KJ (2002). Internalised stigma among people living with HIV/AIDS. AIDS Behav.

[20] Sillers D National and international poverty lines: an overview. Washington, DC: United States Agency for International Development, n.d.:1-12. (http://www.povertyfrontiers.org/ev.php?ID=1075_201&ID2=DO_TOPIC, accessed on 14 January 2012).

[21] Kaiser HF (1960). The application of electronic computers to factor analysis. Educ Psycho Measurement.

[22] Simbayi LC, Kalichman S, Strebel A, Cloete A, Henda N, Mqeketo A (2007). Internalized stigma, discrimination, and depression among men and women living with HIV/AIDS in Capetown, South Africa. Soc Sci Med.

[23] Li L, Lee SJ, Thammawijaya P, Jiraphongsa C, Rotheram-Borus MJ (2009). Stigma, social support, and depression among people living with HIV in Thailand. AIDS Care.

[24] Vanable PA, Carey MP, Blair DC, Littlewood RA (2006). Impact of HIV related stigma on health behabiours and psychological adjustment among HIV positive men and women. AIDS Behav.

[25] Sayles JN, Hays RD, Sarkisian CA, Mahajan AP, Spritzer KL, Cunningham WE (2008). Development and psycho-metric assessment of a multidimensional measure of internalized HIV stigma in a sample of HIV-positive adults. AIDS Behav.

